# Supplementary material for: Fermentation-Derived Metabolites Shape Host Biology to Attenuate Severity of Inflammatory and Metabolic Disease
Source: bioRxiv. 2026 Aug 1:2026.07.30.741534. Preprint. [Version 1] doi: 10.64898/2026.07.30.741534 (PMC13484210; doi:10.64898/2026.07.30.741534)
Supplement: Supplementary file 1 [file NIHPP2026.07.30.741534v1-supplement-1.pdf]

A.

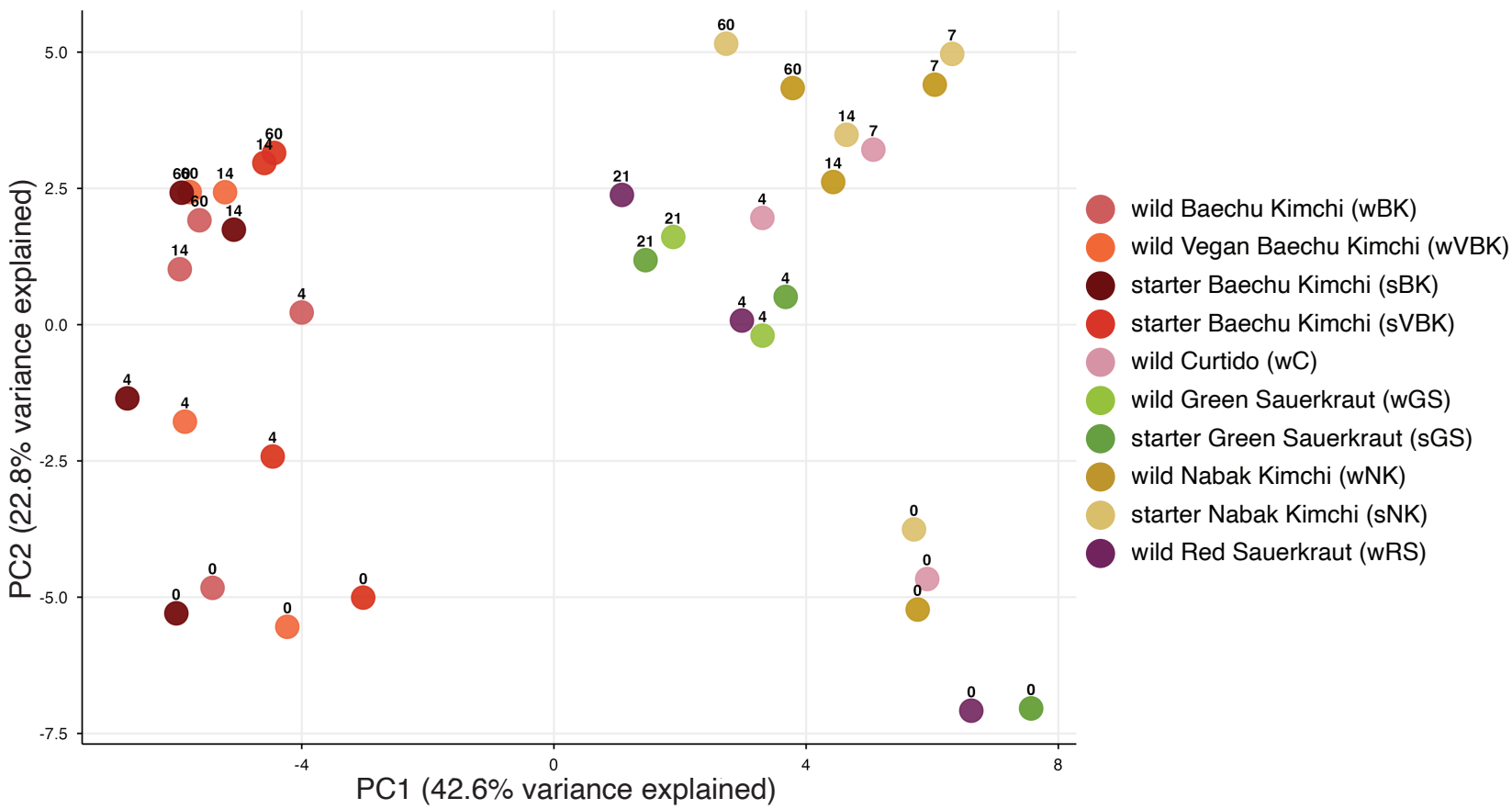

PC2 (22.8% variance explained)
PC1 (42.6% variance explained)
wild Baechu Kimchi (wBK)
wild Vegan Baechu Kimchi (wVBK)
starter Baechu Kimchi (sBK)
starter Baechu Kimchi (sVBK)
wild Curtido (wC)
wild Green Sauerkraut (wGS)
starter Green Sauerkraut (sGS)
wild Nabak Kimchi (wNK)
starter Nabak Kimchi (sNK)
wild Red Sauerkraut (wRS)

B.

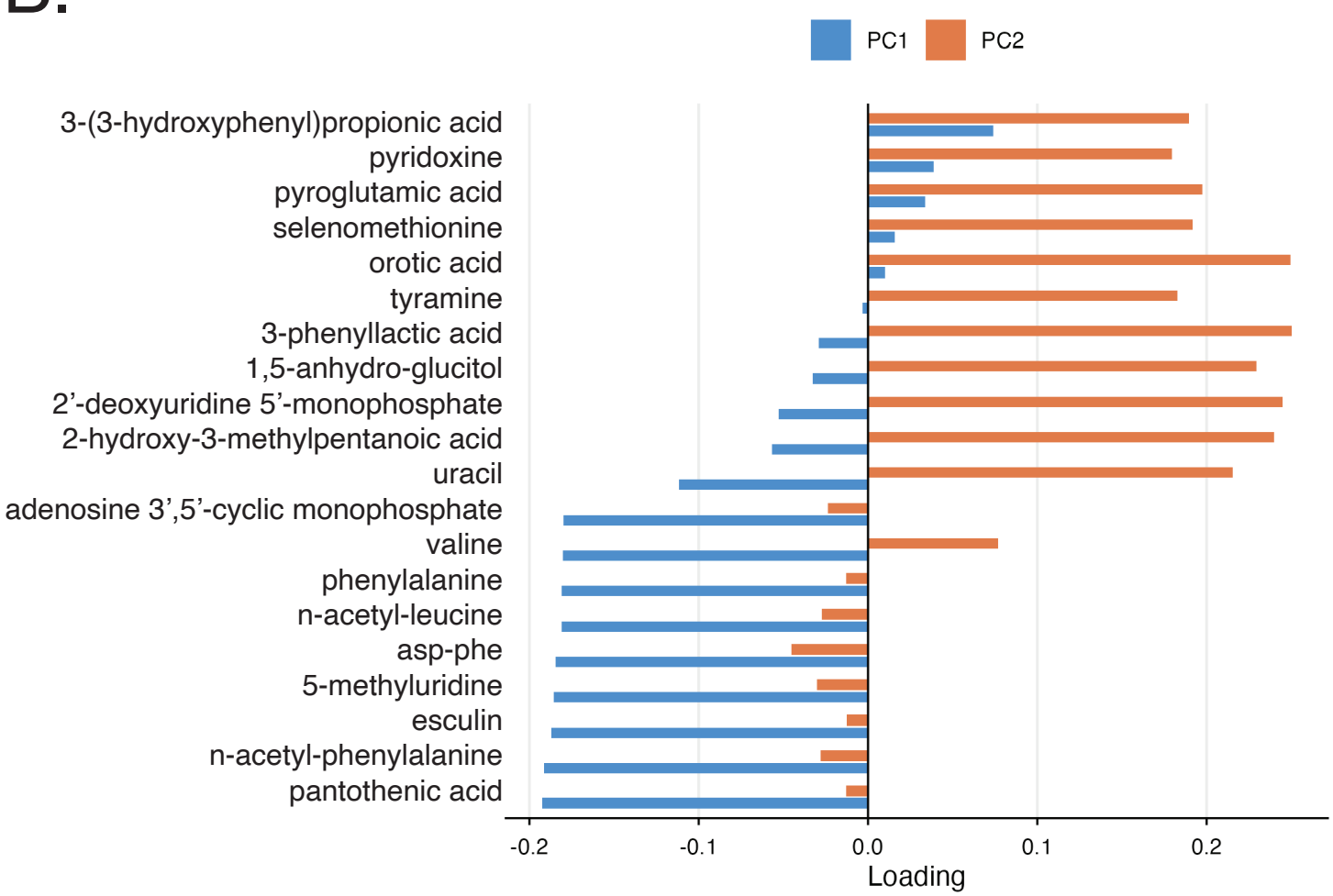

PC1
PC2
3-(3-hydroxyphenyl)propionic acid
pyridoxine
pyroglutamic acid
selenomethionine
orotic acid
tyramine
3-phenyllactic acid
1,5-anhydro-glucitol
2'-deoxyuridine 5'-monophosphate
2-hydroxy-3-methylpentanoic acid
uracil
adenosine 3',5'-cyclic monophosphate
valine
phenylalanine
n-acetyl-leucine
asp-phe
5-methyluridine
esculin
n-acetyl-phenylalanine
pantothenic acid
Loading

bioRxiv preprint doi: https://doi.org/10.64898/2026.07.30.741534; this version posted August 1, 2026. The copyright holder for this preprint (which was not certified by peer review) is the author/funder, who has granted bioRxiv a license to display the preprint in perpetuity. It is made available under a CC-BY 4.0 International license.

Supplementary Figure 1. Metabolomics characterization of vegetable based fermented foods (VBFF). (A) Probabilistic principal component analysis (PPCA) of untargeted metabolomics profiles across all fermented food samples and fermentation stages. Each point represents a biological replicate; colors indicate food and fermentation conditions. PC1 and PC2 explain 42.6% and 22.9% of total variance, respectively. (B) PCA loadings for PC1 and PC2 showing the top metabolites driving sample separation.

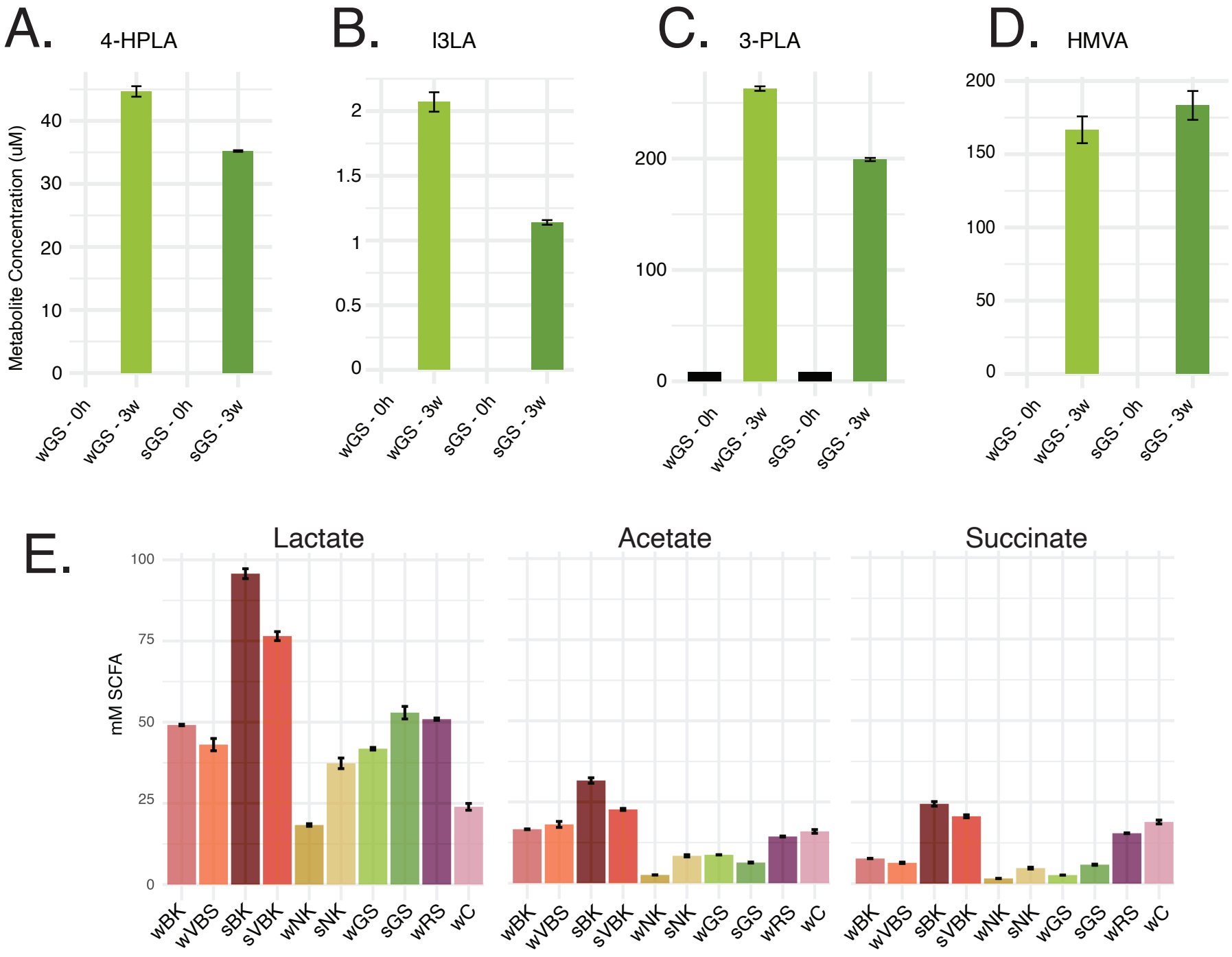

A.
4-HPLA
Metabolite Concentration (uM)
0
10
20
30
40
wGS - 0h
wGS - 3w
sGS - 0h
sGS - 3w
B.
I3LA
0
0.5
1
1.5
2
wGS - 0h
wGS - 3w
sGS - 0h
sGS - 3w
C.
3-PLA
0
100
200
wGS - 0h
wGS - 3w
sGS - 0h
sGS - 3w
D.
HMVA
0
50
100
150
200
wGS - 0h
wGS - 3w
sGS - 0h
sGS - 3w
E.
Lactate
Acetate
Succinate
mM SCFA
0
25
50
75
100
wBK
wVBS
sBK
sVBK
wNK
sNK
wGS
sGS
wRS
wC

bioRxiv preprint doi: https://doi.org/10.64898/2026.07.30.741534; this version posted August 1, 2026. The copyright holder for this preprint (which was not certified by peer review) is the author/funder, who has granted bioRxiv a license to display the preprint in perpetuity. It is made available under a CC-BY 4.0 International license.

Supplementary Figure 2. Targeted quantification of metabolites of interest and short-chain fatty acids (SCFAs). (A) Targeted LC-MS/MS quantification of 4-hydroxyphenyllactic acid (4-HPLA), (B) indole-3-lactic acid (I3LA), (C) 3-phenyllactic acid (3-PLA), (D) and HMVA (μM) in green sauerkraut fermented with (sGS) or without starter culture (wGS) at 0 hours and 3 weeks, validating untargeted metabolomics findings. Values represent mean ± SEM. (See Supplementary Table 2.) (E) SCFA quantification (mM) of lactate, acetate, and succinate across all fermented food conditions at the final fermentation timepoint (3 weeks), confirming active lactic acid fermentation. Values represent mean ± SEM. (See Supplementary Table 2.)

A.

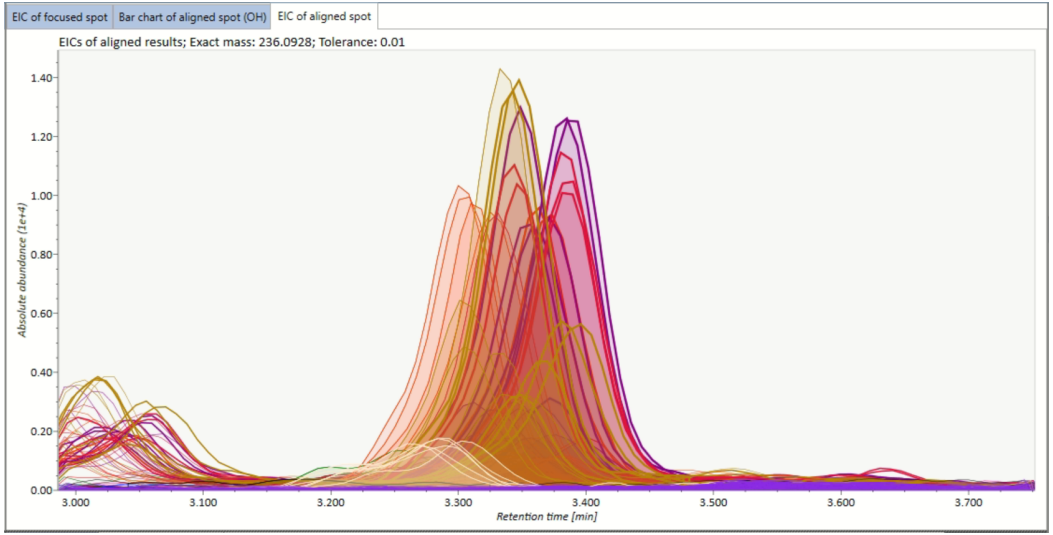

EIC of focused spot
Bar chart of aligned spot (OH)
EIC of aligned spot
EICs of aligned results; Exact mass: 236.0928; Tolerance: 0.01
Absolute abundance (1e+4)
1.40
1.20
1.00
0.80
0.60
0.40
0.20
0.00
3.000
3.100
3.200
3.300
3.400
3.500
3.600
3.700
Retention time [min]

B.

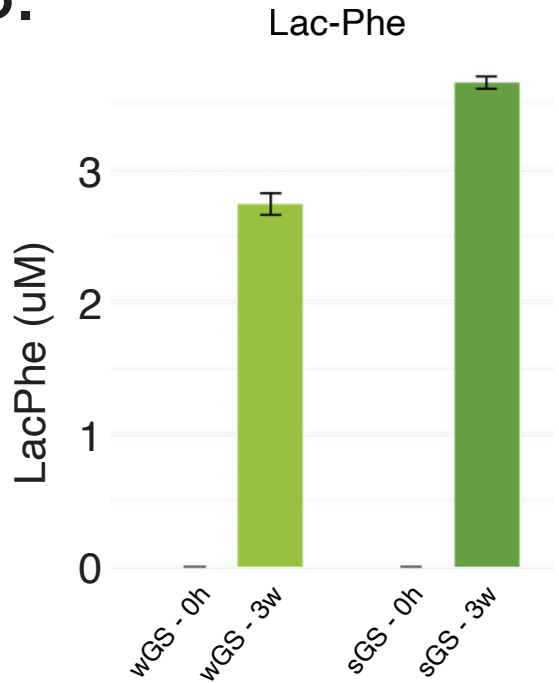

Lac-Phe
LacPhe (uM)
0
1
2
3
wGS - 0h
wGS - 3w
sGS - 0h
sGS - 3w

bioRxiv preprint doi: https://doi.org/10.64898/2026.07.30.741534; this version posted August 1, 2026. The copyright holder for this preprint (which was not certified by peer review) is the author/funder, who has granted bioRxiv a license to display the preprint in perpetuity. It is made available under a CC-BY 4.0 International license.

Supplementary Figure 3. Identification and targeted quantification of Lac-Phe (A) Extracted ion chromatograms from MS-DIAL of mass-to-charge ratio (m/z) corresponding to N-lactoyl-phenylalanine (Lac-Phe) (m/z = 236.0928, negative mode) across all fermented food samples at multiple fermentation stages. (B) Lac-Phe concentrations measured by targeted LC-MS/MS in Green Sauerkraut fermented with (sGS) or without starter culture (wGS) at 0 hours and 3 weeks. Lac-Phe was quantified using an authentic synthetic standard and confirmed by matching retention time and Multiple Reaction Monitoring (MRM) transition (236.1→147 m/z, 236.1→88.1 m/z). Values represent mean ± SEM.

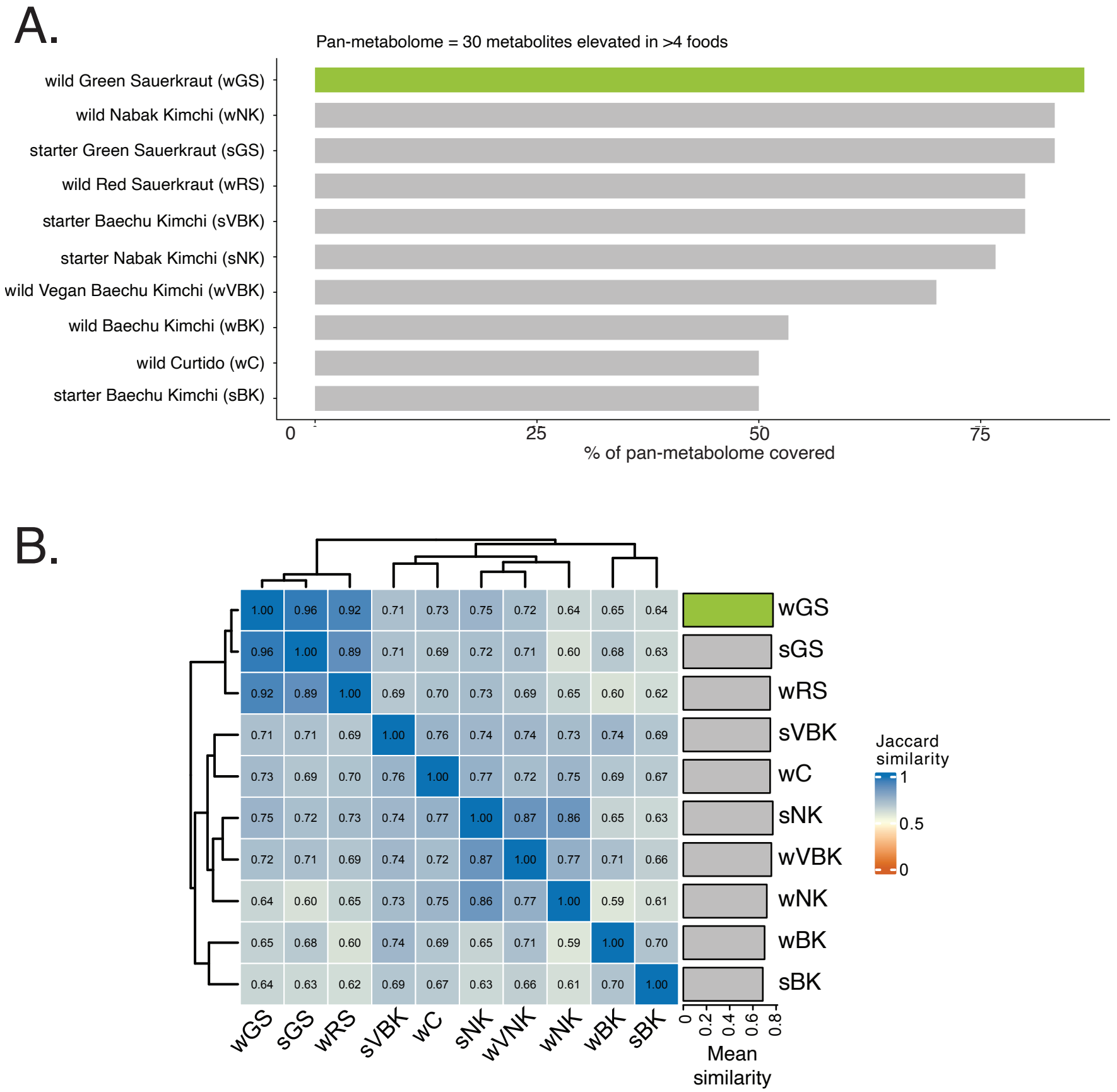
A.
Pan-metabolome = 30 metabolites elevated in >4 foods
wild Green Sauerkraut (wGS)
wild Nabak Kimchi (wNK)
starter Green Sauerkraut (sGS)
wild Red Sauerkraut (wRS)
starter Baechu Kimchi (sVBK)
starter Nabak Kimchi (sNK)
wild Vegan Baechu Kimchi (wVBK)
wild Baechu Kimchi (wBK)
wild Curtido (wC)
starter Baechu Kimchi (sBK)
0
25
50
75
% of pan-metabolome covered
B.
1.00 0.96 0.92 0.71 0.73 0.75 0.72 0.64 0.65 0.64
0.96 1.00 0.89 0.71 0.69 0.72 0.71 0.60 0.68 0.63
0.92 0.89 1.00 0.69 0.70 0.73 0.69 0.65 0.60 0.62
0.71 0.71 0.69 1.00 0.76 0.74 0.74 0.73 0.74 0.69
0.73 0.69 0.70 0.76 1.00 0.77 0.72 0.75 0.69 0.67
0.75 0.72 0.73 0.74 0.77 1.00 0.87 0.86 0.65 0.63
0.72 0.71 0.69 0.74 0.72 0.87 1.00 0.77 0.71 0.66
0.64 0.60 0.65 0.73 0.75 0.86 0.77 1.00 0.59 0.61
0.65 0.68 0.60 0.74 0.69 0.65 0.71 0.59 1.00 0.70
0.64 0.63 0.62 0.69 0.67 0.63 0.66 0.61 0.70 1.00
wGS
sGS
wRS
sVBK
wC
sNK
wVBK
wNK
wBK
sBK
Jaccard similarity
1
0.5
0
0 0.2 0.4 0.6 0.8
Mean similarity

bioRxiv preprint doi: https://doi.org/10.64898/2026.07.30.741534; this version posted August 1, 2026. The copyright holder for this preprint (which was not certified by peer review) is the author/funder, who has granted bioRxiv a license to display the preprint in perpetuity. It is made available under a CC-BY 4.0 International license.

Supplementary Figure 4. Pan-metabolome coverage. (A) The pan-fermented-food metabolome was defined as metabolites enriched ($log_2FC > 2$) in at least 4 fermented foods. Bars show the percentage of pan-metabolome metabolites covered by each condition. Green Sauerkraut (wGS; highlighted) showed the highest coverage and was selected as the primary food model. (B) Jaccard similarity heatmap across all fermented food conditions based on shared upregulated metabolites. Values represent pairwise similarity scores (0 = no overlap, 1 = identical); clustering was performed using Euclidean distance. Bar annotation (right) shows mean similarity per food across all others.

bioRxiv preprint doi: https://doi.org/10.64898/2026.07.30.741534; this version posted August 1, 2026. The copyright holder for this preprint (which was not certified by peer review) is the author/funder, who has granted bioRxiv a license to display the preprint in perpetuity. It is made available under a CC-BY 4.0 International license.

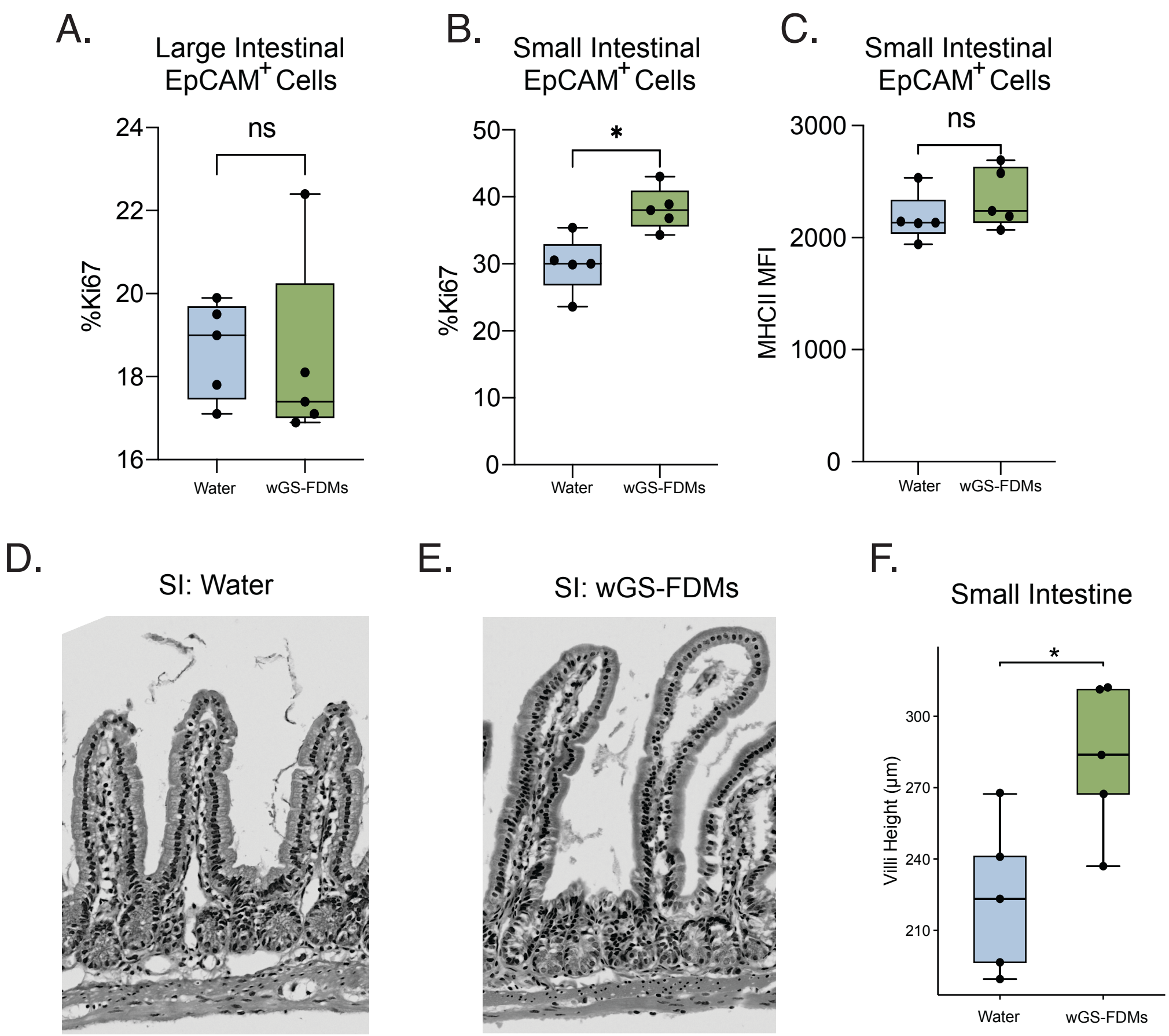

bioRxiv preprint doi: https://doi.org/10.64898/2026.07.30.741534; this version posted August 1, 2026. The copyright holder for this preprint (which was not certified by peer review) is the author/funder, who has granted bioRxiv a license to display the preprint in perpetuity. It is made available under a CC-BY 4.0 International license.

Supplemental Figure 5. wGS-FDMs promote small intestinal epithelial growth without altering immune activation. (A) Percent of $Ki67^+$ proliferating cells among $EpCAM^+$ in large intestinal or (B) small intestinal epithelial cells for water and wGS-FDM-treated mice. wGS-FDM treatment was associated with increased epithelial proliferation in the small intestine, but not in the large intestine. (C) Major histocompatibility complex class II (MHCII) expression (mean fluorescence intensity (MFI)) on $EpCAM^+$ small intestinal epithelial cells from water and wGS-FDM-treated mice. (D–E) Representative hematoxylin and eosin (H&E)-stained sections of the small intestine from water-treated and wGS-FDM-treated mice, showing villus morphology. (F) Quantification of villus height (μm) in the small intestine of water and wGS-FDM-treated mice. Data are presented as box-and-whisker plots with individual data points overlaid. Statistical significance was determined by Mann-Whitney U test or unpaired Student's t-test. *$p < 0.05$; ns, not significant. n = 5–7 mice per group.

Lamina Propria Flow Gating Strategy

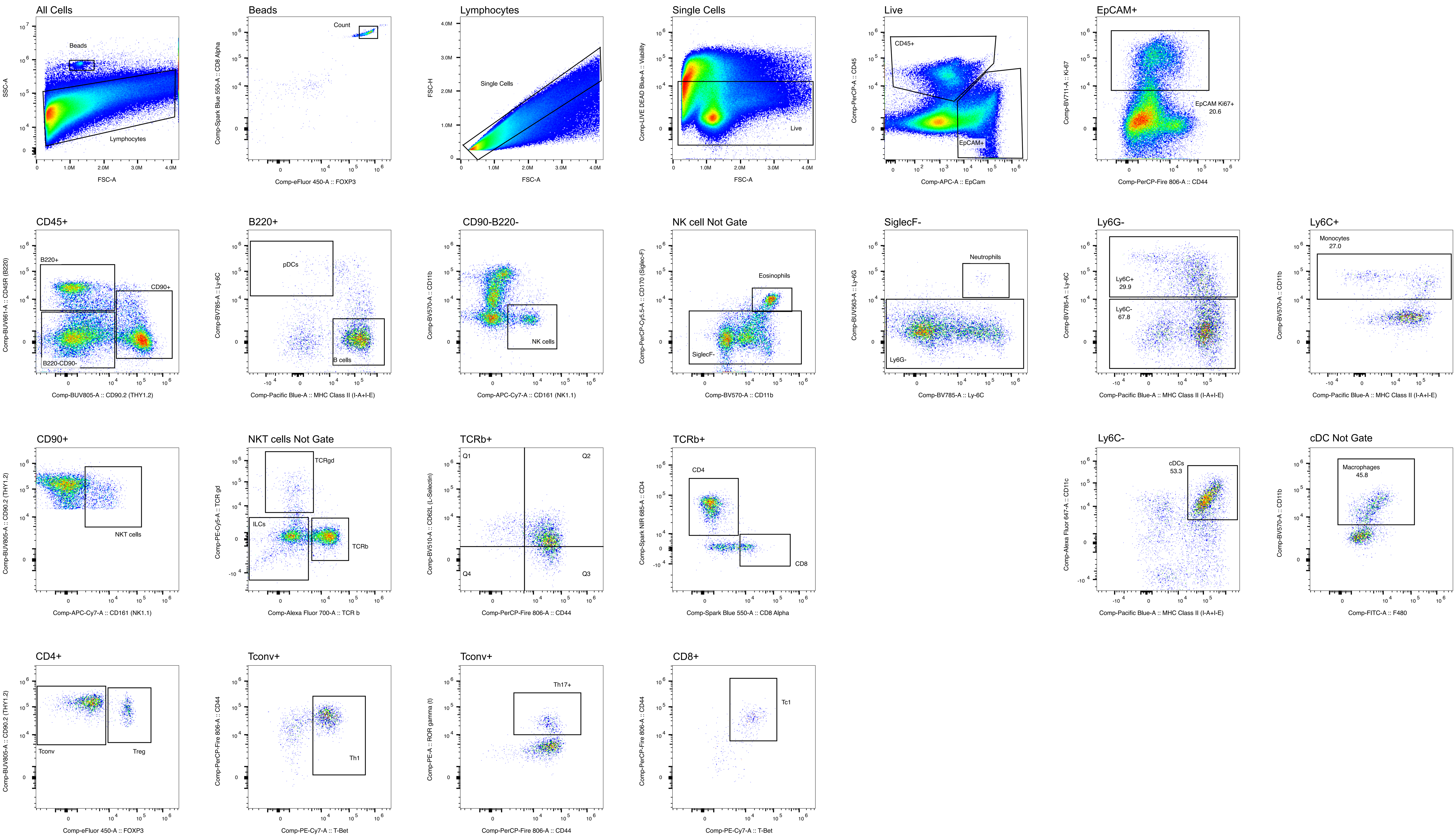

Spleen Flow Gating Strategy

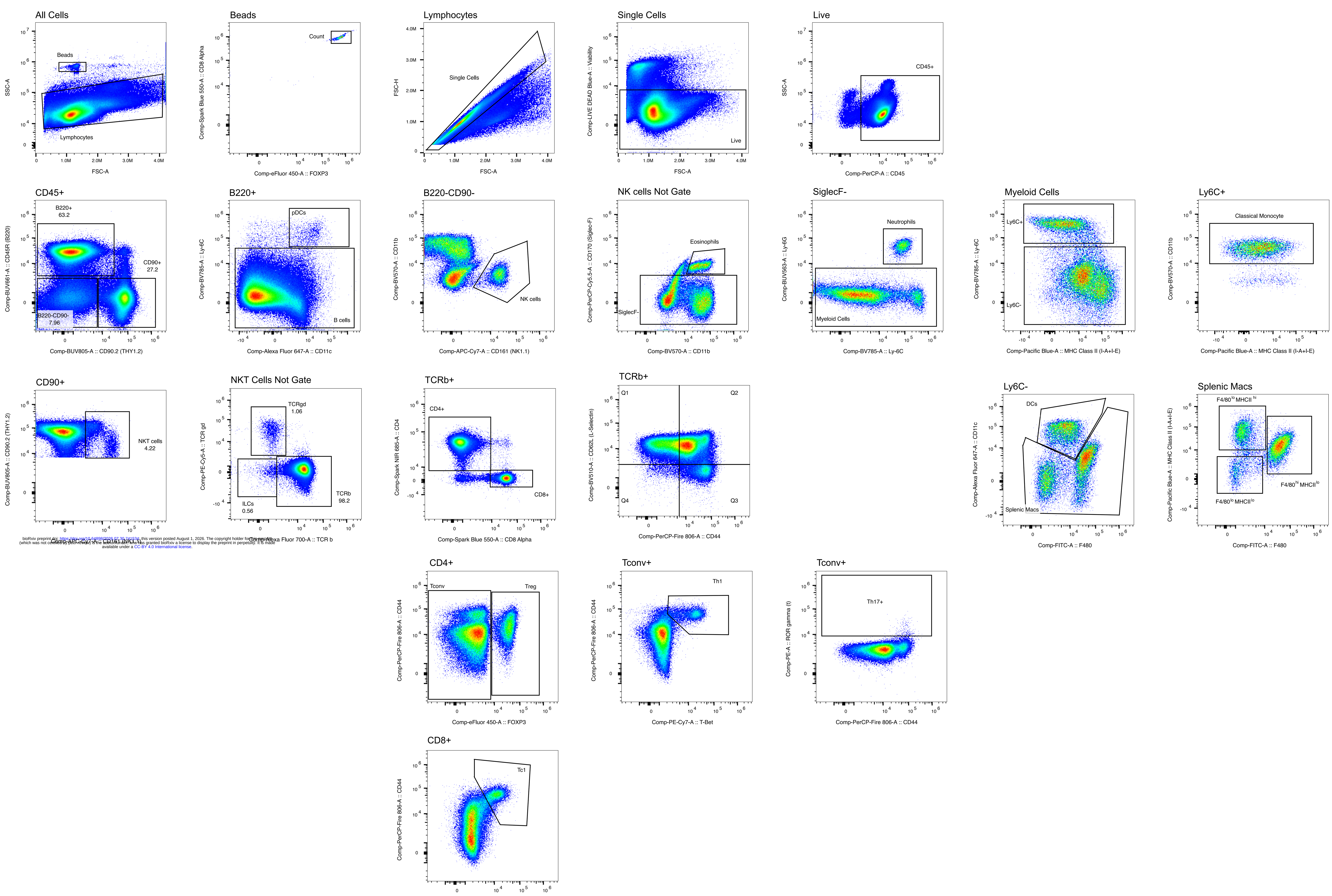

available under a CC-BY 4.0 International license.

bioRxiv preprint doi: https://doi.org/10.64898/2026.07.30.741534; this version posted August 1, 2026. The copyright holder for this preprint (which was not certified by peer review) is the author/funder, who has granted bioRxiv a license to display the preprint in perpetuity. It is made available under a CC-BY 4.0 International license.

Supplemental Figure 6. Gating strategy for spleen and lamina propria. Representative gating plots showing the sequential gating hierarchy used to identify immune populations in the small intestinal lamina propria (SI-LP), large intestinal lamina propria (LI-LP), and spleen.

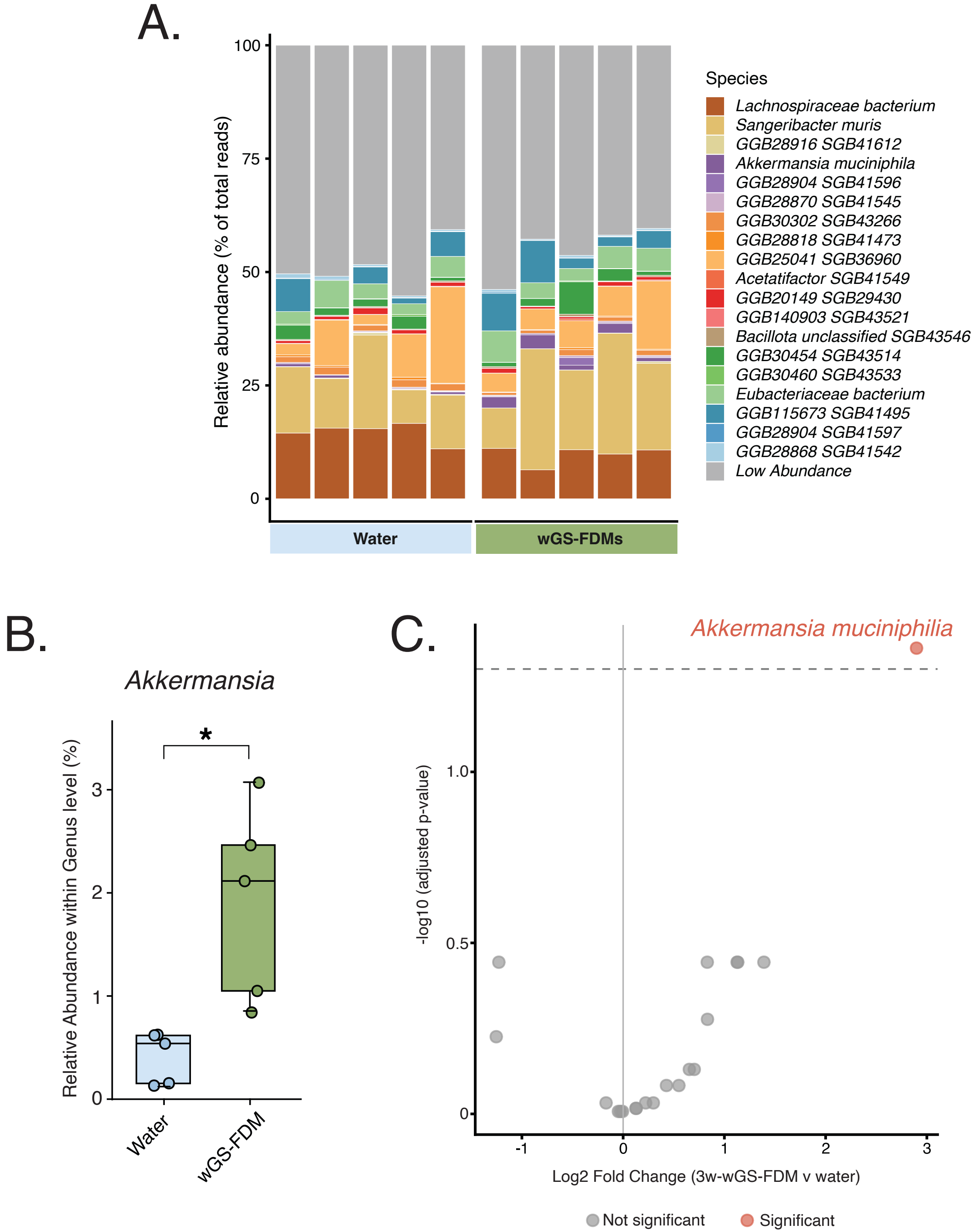
A.
Relative abundance (% of total reads)
100
75
50
25
0
Water
wGS-FDMs
Species
Lachnospiraceae bacterium
Sangeribacter muris
GGB28916 SGB41612
Akkermansia muciniphila
GGB28904 SGB41596
GGB28870 SGB41545
GGB30302 SGB43266
GGB28818 SGB41473
GGB25041 SGB36960
Acetatifactor SGB41549
GGB20149 SGB29430
GGB140903 SGB43521
Bacillota unclassified SGB43546
GGB30454 SGB43514
GGB30460 SGB43533
Eubacteriaceae bacterium
GGB115673 SGB41495
GGB28904 SGB41597
GGB28868 SGB41542
Low Abundance
B.
Akkermansia
*
Relative Abundance within Genus level (%)
3
2
1
0
Water
wGS-FDM
C.
Akkermansia muciniphilia
-log10 (adjusted p-value)
1.0
0.5
0
-1
0
1
2
3
Log2 Fold Change (3w-wGS-FDM v water)
Not significant
Significant

bioRxiv preprint doi: https://doi.org/10.64898/2026.07.30.741534; this version posted August 1, 2026. The copyright holder for this preprint (which was not certified by peer review) is the author/funder, who has granted bioRxiv a license to display the preprint in perpetuity. It is made available under a CC-BY 4.0 International license.

Supplemental Figure 7. Impact of wGS-FDMs on microbiome composition and *Akkermansia* relative abundance. (A) Relative abundance of microbial species in cecal content of water and wGS-FDM-treated mice (n = 5 per group), with taxa contributing less than a defined threshold (<1%) grouped as 'Low Abundance'. (B) Genus-level Mann-Whitney U test for genus *Akkermansia* (BH-adjusted p-value). (C) Differential abundance comparing mice treated with water or 3w-wGS-FDMs (BH-adjusted).

bioRxiv preprint doi: https://doi.org/10.64898/2026.07.30.741534; this version posted August 1, 2026. The copyright holder for this preprint (which was not certified by peer review) is the author/funder, who has granted bioRxiv a license to display the preprint in perpetuity. It is made available under a CC-BY 4.0 International license.

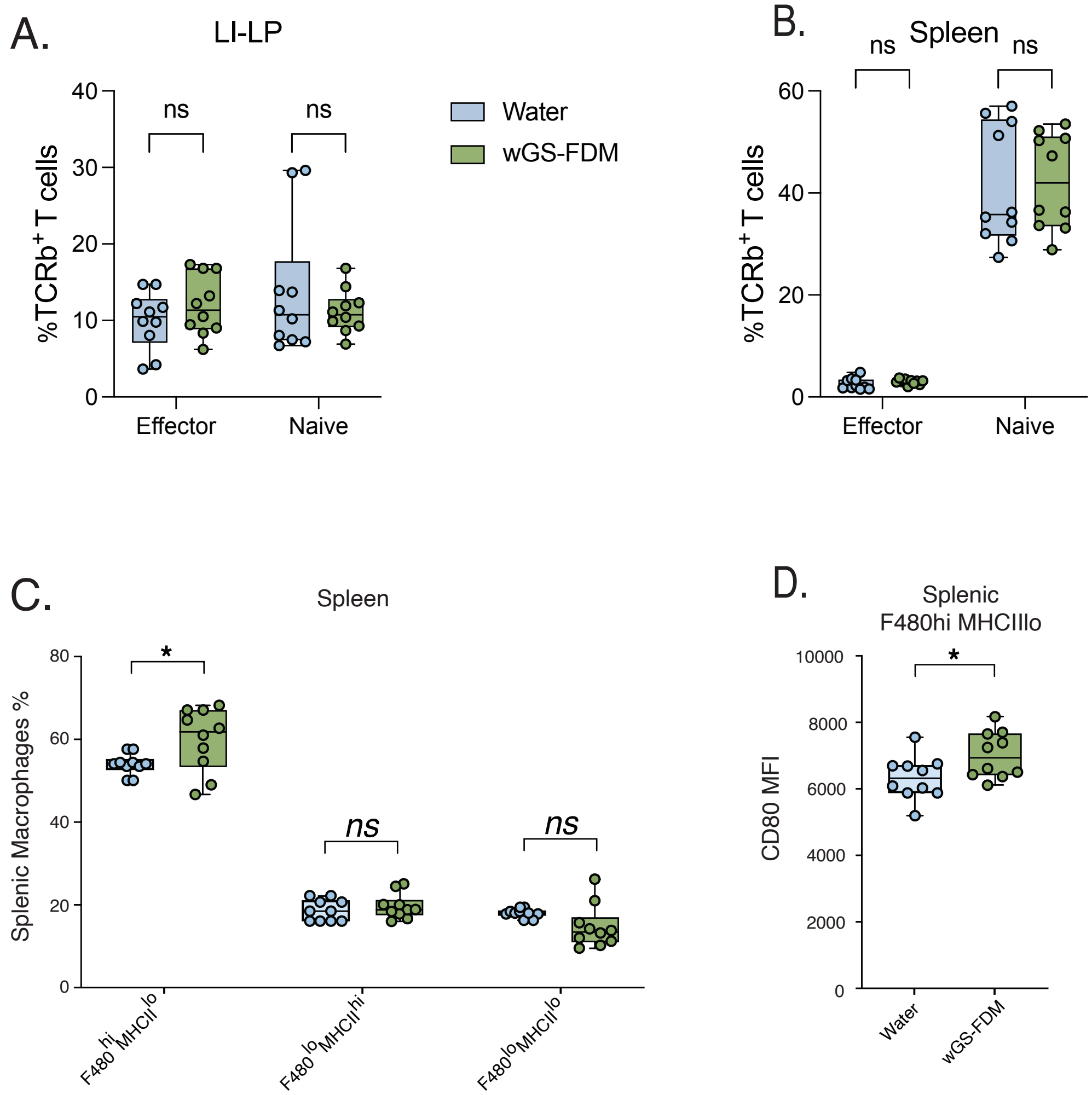

bioRxiv preprint doi: https://doi.org/10.64898/2026.07.30.741534; this version posted August 1, 2026. The copyright holder for this preprint (which was not certified by peer review) is the author/funder, who has granted bioRxiv a license to display the preprint in perpetuity. It is made available under a CC-BY 4.0 International license.

Supplemental Figure 8. wGS-FDM treatment does not affect T cell proportions but increases splenic F480^{hi}MHCII^{lo} macrophage frequency and CD80 expression. (A–B) Frequency of TCRβ^{+} T cells among total live cells in (A) the large intestinal lamina propria (LI-LP) and (B) spleen, gated on effector and naïve T cell populations. (C) Frequency of splenic macrophage subsets — F480^{hi}MHCII^{lo}, F480^{lo}MHCII^{hi}, and F480^{lo}MHCII^{lo} — as a percentage of total splenic macrophages in water- and 3w SK-treated mice. (D) CD80 mean fluorescence intensity (MFI) on splenic F4/80^{hi}MHCII^{lo} macrophages from water- and wGS-FDM-treated mice. Mice received either water (blue) or 3-week SK treatment (3w SK, green). Each dot represents an individual mouse. Boxes indicate interquartile range with median; whiskers show min/max values. *, $p < 0.05$; ns, not significant (unpaired t-test or Mann-Whitney test). Data are pooled from 2 independent experiments with n = 10 mice per group.

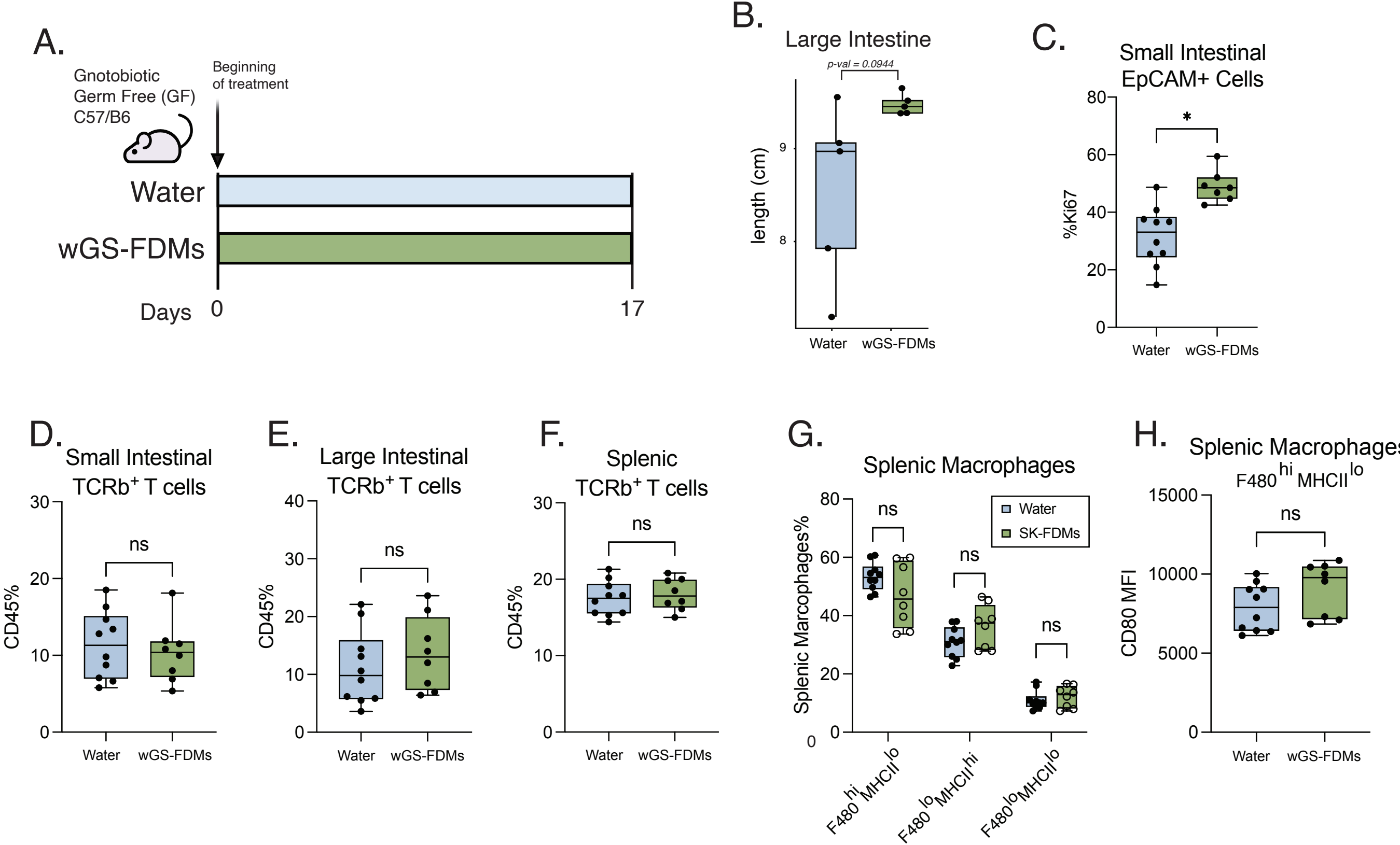

A.
Gnotobiotic
Germ Free (GF)
C57/B6
Beginning
of treatment
Water
wGS-FDMs
Days 0 17
B.
Large Intestine
p-val = 0.0944
length (cm)
Water wGS-FDMs
C.
Small Intestinal
EpCAM+ Cells
%Ki67
*
Water wGS-FDMs
D.
Small Intestinal
TCRb+ T cells
CD45%
ns
Water wGS-FDMs
E.
Large Intestinal
TCRb+ T cells
CD45%
ns
Water wGS-FDMs
F.
Splenic
TCRb+ T cells
CD45%
ns
Water wGS-FDMs
G.
Splenic Macrophages
Splenic Marcophages%
Water
SK-FDMs
ns
F480hi MHCIIlo
F480lo MHCIIhi
F480lo MHCIIlo
H.
Splenic Macrophages
F480hi MHCIIlo
CD80 MFI
ns
Water wGS-FDMs

bioRxiv preprint doi: https://doi.org/10.64898/2026.07.30.741534; this version posted August 1, 2026. The copyright holder for this preprint (which was not certified by peer review) is the author/funder, who has granted bioRxiv a license to display the preprint in perpetuity. It is made available under a CC-BY 4.0 International license.

Supplemental Figure 9. wGS-FDMs promote intestinal epithelial proliferation without altering T cell or macrophage populations in germ-free mice. (A) Schematic of the experimental design. Germ-free (GF) C57BL/6 mice were administered water or wGS-FDMs daily from day 1 through day 17. (B) Large intestine length (cm) of water and wGS-FDM-treated GF mice at endpoint (day 17) ($p = 0.0944$). (C) Percent of Ki67^{+} cells among EpCAM^{+} small intestinal epithelial cells in water and wGS-FDM-treated mice. (D) Percent of TCRβ^{+} T cells among CD45^{+} cells in the small intestine lamina propria, (E) large intestine lamina propria, and (F) spleen of water and wGS-FDM-treated mice. (G) Proportions of splenic macrophage subsets — F480^{hi}MHCII^{lo}, F480^{lo}MHCII^{hi}, and F480^{lo}MHCII^{lo} — as a percentage of total splenic macrophages in water and wGS-FDM-treated mice. (H) CD80 expression (MFI) on F480^{hi}MHCII^{lo} splenic macrophages from water and wGS-FDM-treated mice. Data are presented as box-and-whisker plots (median ± interquartile range, with whiskers extending to minimum and maximum values) with individual data points overlaid. Statistical comparisons were performed using a two-tailed Mann–Whitney U test or unpaired Student's t-test, as appropriate. *$p < 0.05$; ns, not significant. n = 6–10 mice per group.

bioRxiv preprint doi: https://doi.org/10.64898/2026.07.30.741534; this version posted August 1, 2026. The copyright holder for this preprint (which was not certified by peer review) is the author/funder, who has granted bioRxiv a license to display the preprint in perpetuity. It is made available under a CC-BY 4.0 International license.

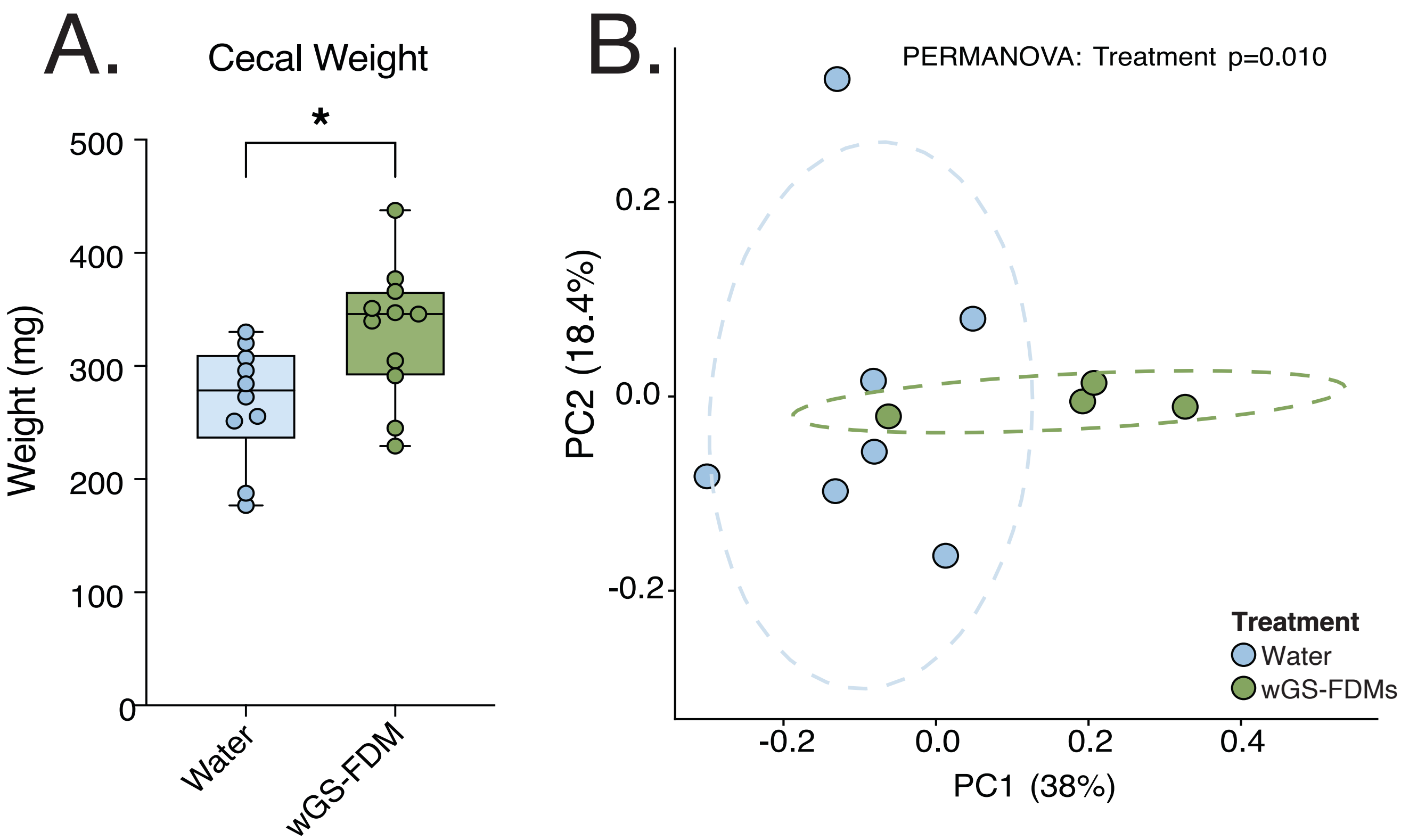

bioRxiv preprint doi: https://doi.org/10.64898/2026.07.30.741534; this version posted August 1, 2026. The copyright holder for this preprint (which was not certified by peer review) is the author/funder, who has granted bioRxiv a license to display the preprint in perpetuity. It is made available under a CC-BY 4.0 International license.

Supplemental Figure 10. wGS-FDM treatment, in DSS colitis, increases cecal weight and shapes a distinct gut microbial community. (A) Cecal weight (mg) of water and wGS-FDM-treated mice at the final timepoint (day 17). Data are presented as box-and-whisker plots (median ± interquartile range, with whiskers extending to minimum and maximum values) with individual data points overlaid. (B) Principal coordinates analysis (PCoA) of Bray-Curtis dissimilarity of gut microbial communities from water and wGS-FDM-treated mice at the final timepoint (day 17). Each point represents an individual mouse. Dashed ellipses indicate 95% confidence intervals for each treatment group. Treatment groups were significantly separated along PC1 (38% variance explained) and PC2 (18.4% variance explained) (PERMANOVA: $F = 2.0021$, $R^2 = 0.143$, $p = 0.010$, 999 permutations). Statistical comparison was performed using an unpaired Student's t-test. *$p < 0.05$. n = 10–13 mice per group.

bioRxiv preprint doi: https://doi.org/10.64898/2026.07.30.741534; this version posted August 1, 2026. The copyright holder for this preprint (which was not certified by peer review) is the author/funder, who has granted bioRxiv a license to display the preprint in perpetuity. It is made available under a CC-BY 4.0 International license.

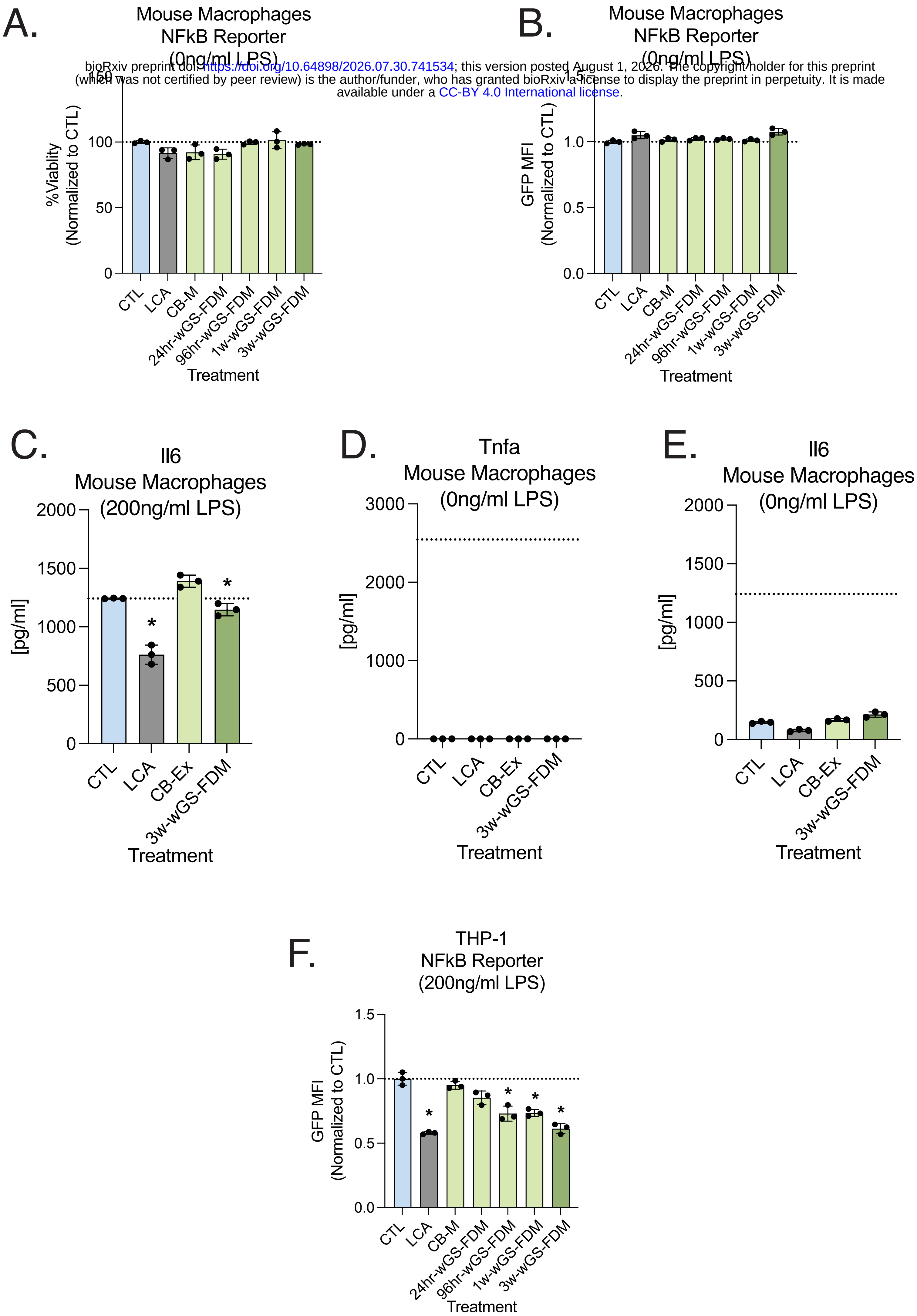

bioRxiv preprint doi: https://doi.org/10.64898/2026.07.30.741534; this version posted August 1, 2026. The copyright holder for this preprint (which was not certified by peer review) is the author/funder, who has granted bioRxiv a license to display the preprint in perpetuity. It is made available under a CC-BY 4.0 International license.

Supplemental Figure 11. wGS-FDMs suppress NFκB activity and IL-6 secretion in LPS-stimulated macrophages without affecting cell viability or basal inflammatory tone. (A) Percent viability of mouse macrophages expressing an NFκB–GFP reporter, normalized to control (CTL), following treatment with lithocholic acid (LCA), cabbage metabolites (CB-M), 24hr-wGS-FDM, 96hr-wGS-FDM, 1w-wGS-FDM, or 3w-wGS-FDM in the absence of lipopolysaccharide (LPS). (B) NFκB activity, measured as GFP MFI normalized to CTL, in mouse macrophage NFκB reporter cells treated as in (A) under basal conditions (0 ng/ml LPS). (C) IL-6 secretion (pg/ml) by mouse macrophages stimulated with LPS (200 ng/ml) and treated with CTL, LCA, CB-Ex, or 3w-wGS-FDM. (D) TNF-α secretion (pg/ml) by mouse macrophages treated with CTL, LCA, CB-Ex, or 3w-wGS-FDM under basal conditions (0 ng/ml LPS). (E) IL-6 secretion (pg/ml) by mouse macrophages treated with CTL, LCA, CB-Ex, or 3w-wGS-FDM under basal conditions (0 ng/ml LPS). (F) NFκB activity (GFP MFI normalized to CTL) in THP-1 NFκB reporter cells stimulated with LPS (200 ng/ml) and treated with LCA, CB-M, 24hr-wGS-FDM, 96hr-wGS-FDM, 1w-wGS-FDM, or 3w-wGS-FDM. Data are presented as mean ± SD with individual data points overlaid. Dashed lines indicate the CTL reference value. Statistical comparisons were performed using one-way ANOVA with Dunnett's post hoc test or a Kruskal–Wallis test with Dunn's correction, as appropriate, comparing each treatment to CTL. *$p < 0.05$. $n = 3–5$ independent experiments per group.

bioRxiv preprint doi: https://doi.org/10.64898/2026.07.30.741534; this version posted August 1, 2026. The copyright holder for this preprint (which was not certified by peer review) is the author/funder, who has granted bioRxiv a license to display the preprint in perpetuity. It is made available under a CC-BY 4.0 International license.

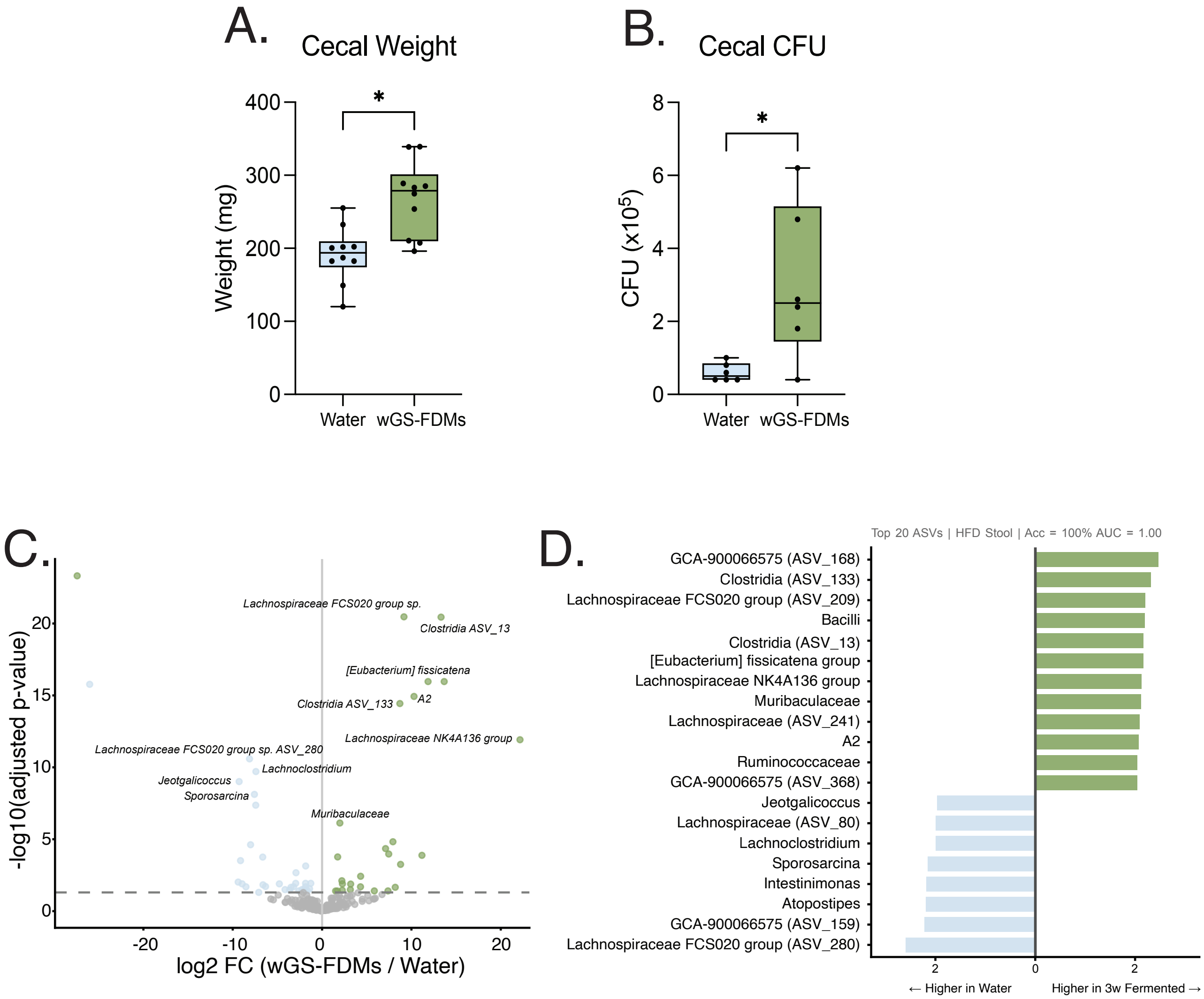

bioRxiv preprint doi: https://doi.org/10.64898/2026.07.30.741534; this version posted August 1, 2026. The copyright holder for this preprint (which was not certified by peer review) is the author/funder, who has granted bioRxiv a license to display the preprint in perpetuity. It is made available under a CC-BY 4.0 International license.

Supplemental Figure 12. wGS-FDM treatment increases cecal bacterial load and enriches distinct microbial taxa in HFD mice. (A) Cecal weight (mg) of water and wGS-FDM-treated mice at the final timepoint (week 10) presented as box-and-whisker plots (median ± interquartile range, with whiskers extending to minimum and maximum values) with individual data points overlaid. (B) Cecal bacterial burden, quantified as colony-forming units (CFU × 10^5), from water and wGS-FDM-treated mice presented as box-and-whisker plots as described in (A). (C) Volcano plot depicting differentially abundant amplicon sequence variants (ASVs) between wGS-FDM- and water-treated mice over weeks of treatment. The x-axis shows log2 fold change (wGS-FDMs / Water) and the y-axis shows −log10(adjusted p-value). The dashed horizontal line indicates the significance threshold (adjusted $p < 0.05$). Green points represent ASVs significantly enriched in wGS-FDM-treated mice; blue points represent ASVs enriched in water-treated mice. Select taxa are labeled. Differential abundance analysis was performed using DESeq2 with Benjamini–Hochberg correction for multiple comparisons. (D) Horizontal bar plot of the top 20 ASVs identified in high-fat diet (HFD) stool, ranked by a classifier with 100% accuracy (AUC = 1.00). Green bars indicate ASVs more abundant in 3-week fermented wGS-FDM-treated mice; blue bars indicate ASVs more abundant in Water-treated mice. Statistical comparisons were performed using an unpaired Student's t-test. *$p < 0.05$. n = 8–10 mice per group.

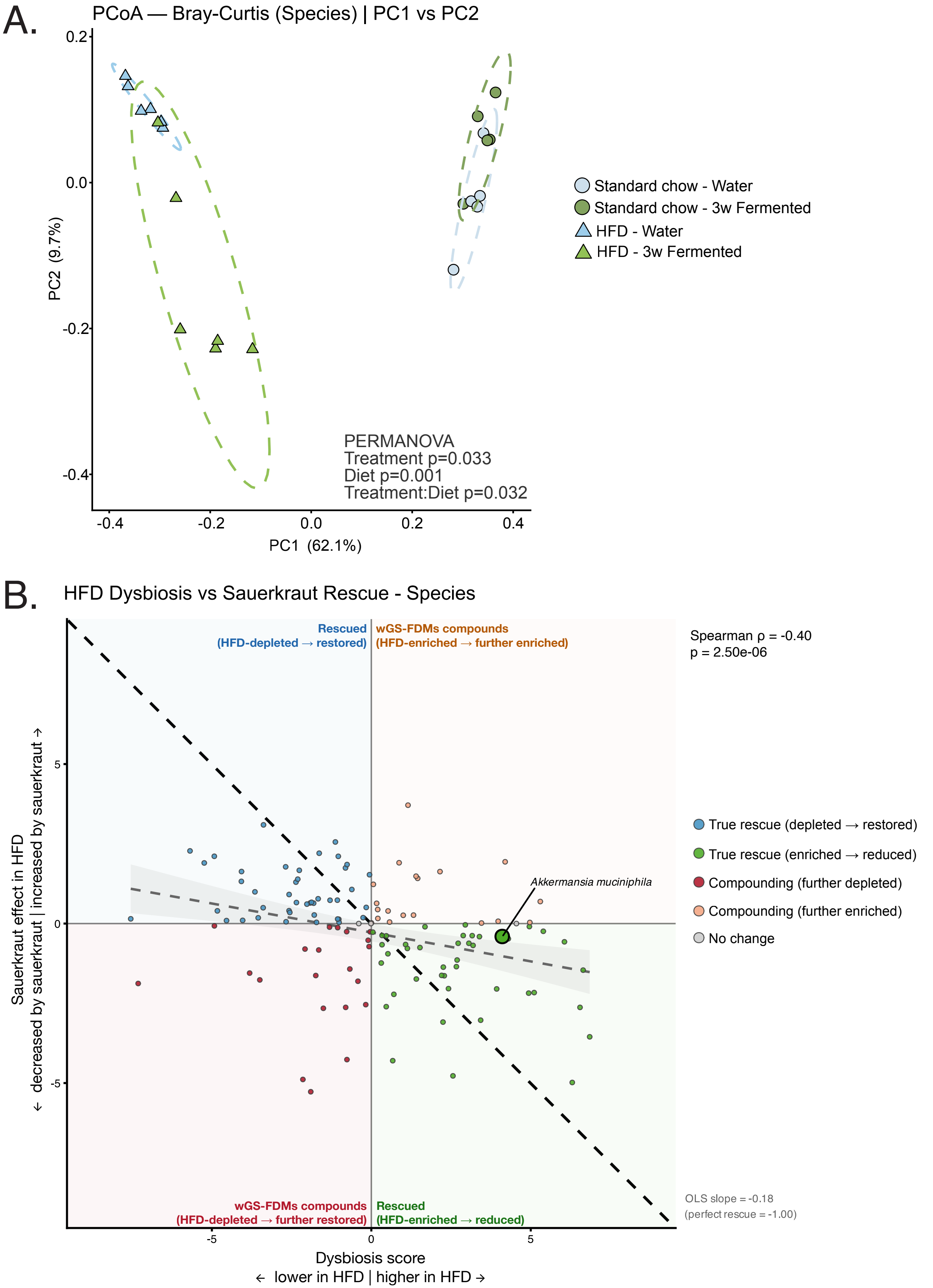

A.
PCoA — Bray-Curtis (Species) | PC1 vs PC2
0.2
0.0
-0.2
-0.4
PC2 (9.7%)
-0.4
-0.2
0.0
0.2
0.4
PC1 (62.1%)
PERMANOVA
Treatment p=0.033
Diet p=0.001
Treatment:Diet p=0.032
Standard chow - Water
Standard chow - 3w Fermented
HFD - Water
HFD - 3w Fermented
B.
HFD Dysbiosis vs Sauerkraut Rescue - Species
Rescued
(HFD-depleted → restored)
wGS-FDMs compounds
(HFD-enriched → further enriched)
Spearman ρ = -0.40
p = 2.50e-06
Akkermansia muciniphila
True rescue (depleted → restored)
True rescue (enriched → reduced)
Compounding (further depleted)
Compounding (further enriched)
No change
5
0
-5
Sauerkraut effect in HFD
← decreased by sauerkraut | increased by sauerkraut →
wGS-FDMs compounds
(HFD-depleted → further restored)
Rescued
(HFD-enriched → reduced)
OLS slope = -0.18
(perfect rescue = -1.00)
-5
0
5
Dysbiosis score
← lower in HFD | higher in HFD →

bioRxiv preprint doi: https://doi.org/10.64898/2026.07.30.741534; this version posted August 1, 2026. The copyright holder for this preprint (which was not certified by peer review) is the author/funder, who has granted bioRxiv a license to display the preprint in perpetuity. It is made available under a CC-BY 4.0 International license.

Supplemental Figure 13. wGS-FDM treatment shifts gut microbiome. (A) Principal coordinates analysis (PCoA) of Bray-Curtis dissimilarity of gut microbial communities from baseline and HFD mice treated with wGS-FDM or untreated at the final timepoint. PERMANOVA revealed significant effects of both diet ($p = 0.001$) and treatment ($p = 0.032$). (B) Comparison of HFD-driven dysbiosis score to sauerkraut effect in HFD model. The x-axis indicated the log2 fold-change of HFD water compared to the baseline water group. The y-axis indicated the log2 fold-change of HFD wGS-FDM treatment to HFD water treatment. The black dashed line represents a perfect rescue, while the grey dashed line indicates the observed OLD trend (OLS slope = -0.18). (Spearman $\rho = -0.040$, p-value = 2.50e-06).
